# Translational Research in Oral Lichen Planus: From Laboratory Discoveries to Clinical Applications

**DOI:** 10.7759/cureus.71462

**Published:** 2024-10-14

**Authors:** Tejaswini Chintha, Priyadharshini B, Aravindhan R, Magesh K T, Swaathi R

**Affiliations:** 1 Oral Pathology and Microbiology, SRM Kattankulathur Dental College and Hospital, SRM Institute of Science and Technology (SRMIST), Chennai, IND

**Keywords:** corticosteroids, dental management, future management, oral lichen planus, photodynamic therapy, treatment choices

## Abstract

Oral lichen planus (OLP) is a chronic inflammatory condition that affects the oral mucous membrane, causing discomfort and pain for millions of people worldwide. Challenges in managing OLP include its chronic nature, varied clinical manifestations, poor patient compliance, and potential for malignant transformation. Recent breakthroughs in the therapy of OLP have opened up new paths for dental practitioners, yet persistent difficulties require attention. Topical calcineurin inhibitors, photodynamic treatment, and biologics have all demonstrated success in relieving symptoms and delaying disease progression. Furthermore, advances in understanding OLP's immunopathogenesis have revealed new targets for targeted treatments, raising hopes for more tailored therapy methods. However, obstacles remain, particularly in the area of long-term treatment and the danger of malignant transformation, needing close monitoring and interdisciplinary teamwork. This review will attempt to explore all of the therapeutic strategies for treating OLP that have been addressed in the literature.

## Introduction and background

Lichen planus is a persistent inflammatory dermatological condition that impacts the skin, hair follicles, scalp, nails, and mucosa of the oral cavity. Unlike cutaneous lichen planus, which is typically self-limiting, the oral variant is characterized by persistent pruritus and recurrent episodes of exacerbation and remission [1]. Although 2%-5% of the general population is affected by this disease, it is primarily common among the female population and may go undetected for a longer duration [2]. While the precise reason remains uncertain, it is thought to be a complex biological process initiated by multiple factors, including stress; malnutrition; allergies; mechanical, psychological, electrochemical, infectious, and mucous-exciting factors; endocrine disorders; salivary gland disorders; genetic susceptibility; and immune illnesses [3]. Andreasen categorized oral lichen planus (OLP) into six types clinically, which include reticular, papular, plaque-like, atrophic, erosive, and bullous [4]. Clinical manifestations might range from moderate discomfort to excruciating pain, affecting speech and appetite. OLP is regarded as an oral potentially malignant disorder, and given that malignant transformation occurs in 1%-2% of adult cases, prompt diagnosis and adequate management are required [5]. OLP has long been a topic of discussion because of its multifaceted etiology, the likelihood of malignant transformation, and diverse clinical symptoms. The fundamental objective of this review is to provide healthcare practitioners with an insight into the therapeutic possibilities of OLP.

## Review

Treatment modalities

In this article, we have segregated and categorized the different treatment modalities for OLP management based on their application method and mechanism of action.

Topical Treatments

Topically applied corticosteroids are the initial therapeutic approach for OLP due to their anti-inflammatory properties [6]. Commonly used medications include clobetasol propionate, triamcinolone acetonide, dexamethasone, betamethasone, and fluticasone, which are applied as 0.05% ointment two to three times daily, as 0.1% paste two to three times daily, as a mouth rinse (0.5 mg/5 ml) two to three times daily (dissolve 500-mg tablets in water and rinse three to four times daily), as a mouth spray to the lesions up to three or four times a day, or as a cream (0.05%) applied to painful lesions three to four times a day, respectively [7]. The side effect of these treatments is oral candidiasis, which is to be managed with antifungal treatments if needed [8]. These treatments are effective for managing OLP symptoms and improving patient comfort. Immunosuppressive drugs are used topically to manage OLP by reducing the immune response. The commonly used topical immunosuppressants include tacrolimus, which is applied as a 0.1% ointment twice daily. It is highly potent, being 10-100 times more effective than cyclosporine. Side effects include mucosal burning and potential relapse within 12 months [9]. Pimecrolimus is used as a 1% cream applied twice daily. It is effective but can cause similar side effects to tacrolimus [10]. Cyclosporine is used as a mouthwash (100 mg/ml, twice daily) or as a topical adhesive. Side effects include hydrophobicity, unpleasant taste, and gingival hyperplasia [11,12]. These topical applications are effective in managing OLP symptoms but require careful monitoring for side effects. Retinoids, derivatives of vitamin A, are used topically to manage OLP due to their anti-inflammatory properties and ability to reduce the keratinization of epithelial cells. Commonly used topical retinoids include tretinoin, isotretinoin, and fenretinide, which are all applied as a 0.1% gel [13]. The benefits include the reduction of lesions, especially effective in reducing reticular and plaque lesions [13]. The side effects are recurrence. The lesions may recur after discontinuing therapy. These topical retinoids are effective in managing OLP symptoms but require careful monitoring for potential side effects [13].

Systemic Treatments

Corticosteroids are reserved for extensive, multifocal lesions with significant ulceration and chronic OLP unresponsive to topical therapies. Prednisone is commonly used; the recommended dosage is 15-30 mg daily taken orally for two weeks, then tapered down to 5 mg daily and stopped altogether after week three [14]. Immunomodulators are used in treating OLP to suppress the immune response and manage severe cases. The commonly used systemic immunomodulators include methotrexate administered as oral tablets or injections. The dosage is typically 7.5-25 mg once weekly. It is effective in reducing inflammation and immune response [15]. It shows significant improvement in clinical condition and symptom remission in 63%-93% of cases. Dapsone as an oral tablet, usually 50-100 mg, has similar efficacy to topical corticosteroids and tacrolimus. It is effective in managing symptoms and reducing inflammation [16]. Azathioprine is also taken as oral tablets, 1-3 mg/kg/day. It disrupts purine synthesis, reducing T and B lymphocyte proliferation. The side effects include pancytopenia, bone marrow suppression, and liver dysfunction [17]. Systemic retinoids like acitretin and isotretinoin are used for severe OLP cases. They modulate the immune response and reduce keratinization. They are given in the form of oral capsules. However, they can cause significant side effects, including liver toxicity and teratogenicity, requiring careful monitoring [18].

Intralesional Treatments

Intralesional treatments include two weekly doses of triamcinolone acetonide (0.2-0.4 ml of a 10-mg/ml solution) [19]. The local side effects include pain, bleeding, infection, allergic reactions, impaired healing, abscesses, pigmentation changes, and skin atrophy whereas the systemic side effects include increased blood sugar and potential systemic absorption.

Phototherapy

Photodynamic therapy (PDT) uses a photosensitizing chemical that is triggered by light of a certain wavelength, such as methylene blue or 5-aminolevulinic acid (5-ALA). The suitable wavelength determines how deeply the light source penetrates the target tissue [20]. This activation produces reactive oxygen species that target and destroy abnormal cells. Studies have shown that PDT can significantly reduce lesion size, pain, and inflammation in OLP patients [21,22]. It is considered as effective as topical corticosteroids and can be a good alternative for patients who are resistant to steroids or when steroids are contraindicated [23]. PUVA therapy combines the use of psoralen (a photosensitizing agent) with UVA light. Psoralen can be administered orally or topically. In the context of OLP, PUVA therapy has shown effectiveness in reducing symptoms and lesions, but it comes with potential side effects such as nausea and an increased risk of carcinogenicity [23]. Topical application of psoralen is still experimental but shows promise with fewer systemic side effects [24]. In comparison, both PDT and PUVA have shown efficacy in managing OLP, with PDT being comparable to corticosteroids and PUVA being effective but with more side effects. PDT generally has fewer side effects compared to PUVA, which can cause nausea and has a potential risk of carcinogenicity. PDT is non-invasive and can be used topically, while PUVA can be administered both orally and topically [25]. The use of low-intensity lasers to alleviate pain and inflammation, speed up the healing process, and control immunological responses is referred to as low-level laser therapy (LLLT). LLLT has shown promising results in managing symptomatic OLP. It works by emitting light at specific wavelengths, which penetrates tissues and induces photochemical reactions. These reactions can reduce inflammation, promote tissue repair, and alleviate pain [26,27]. A systematic review of studies on LLLT for OLP found that it is effective in reducing lesion size, pain, and inflammation without significant adverse effects. The laser wavelengths used in these studies ranged from 630 to 980 nm, with power outputs between 20 and 300 mW and treatment durations from 10 seconds to 15 minutes [25]. A case report even described the successful treatment of an extensive erosive OLP lesion using a 660-nm diode laser, resulting in significant symptom relief and lesion healing within two weeks [27]. The benefits include a non-invasive therapeutic approach with few to no documented side effects. It can be employed as an alternative to corticosteroids, particularly for those who are resistant to or unable to use steroids [26].

Alternative and Complementary Therapies

Herbal remedies like aloe vera have shown promising results. It has anti-inflammatory properties as it contains compounds that inhibit inflammatory pathways, reducing the release of histamine and leukotrienes. This helps in managing the inflammation associated with OLP [28]. Studies have demonstrated burning pain and discomfort can be reduced using aloe vera. Compared to the placebo group, which exhibited only 4% response, 81% of patients treated with aloe vera gel demonstrated a favorable outcome in a study [29]. It also promotes wound healing through its antimicrobial and antioxidant properties. This can be particularly beneficial for the erosive and ulcerative lesions seen in OLP. It has been found to be a safe alternative treatment with no serious side effects reported. It has also been shown to be more effective than some conventional treatments like triamcinolone acetonide in certain studies [30]. Overall, aloe vera offers a natural and effective option for managing OLP, providing both symptom relief and anti-inflammatory benefits. Multiple papers have demonstrated that curcumin protects the neurological and immunological systems while also acting as an antioxidant, antibacterial, anticarcinogenic, antimutagenic, and antiproliferative [31,32]. Reactive oxygen species, such as hydroxyl radicals and amine superoxide, can be neutralized by it. The antifungal properties of corticosteroids prevent candidiasis, which is one of the most common adverse effects [33]. Even at exceedingly high dosages, it is completely safe. The magnitude of the lesion, erythema, or pain of OLP was not significantly influenced by curcumin in comparison to the control groups. This approach for OLP remains uncertain in terms of its clinical efficacy and safety, necessitating further research [34]. Nutritional supplements like omega-3 polyunsaturated fatty acids (n-3 PUFAs) in individuals have demonstrated the potential to effectively manage OLP. By acting as anti-inflammatory agents, n-3 PUFAs like eicosapentaenoic acid (EPA) and docosahexaenoic acid (DHA) can inhibit inflammatory pathways, including the NF-κB signaling pathway. This helps reduce the inflammation associated with OLP [35]. These fatty acids can modulate the overexpression of inflammatory cytokines, which are crucial in the pathogenesis of OLP. By reducing cytokine levels, n-3 PUFAs help in controlling the disease’s progression [35]. n-3 PUFAs inhibit the expression of matrix metalloproteinase-9 (MMP-9), an enzyme involved in tissue remodeling and inflammation. This inhibition can help in reducing tissue damage in OLP [35]. n-3 PUFAs also regulate the expression of microRNAs (miRNAs) and autophagy, which are involved in the cellular mechanisms underlying OLP [35]. These fatty acids may ameliorate psychological disorders and oxidative damage, both of which are implicated in the exacerbation of OLP [35]. Overall, n-3 PUFAs offer a non-toxic, cost-effective approach to managing OLP, providing anti-inflammatory and protective benefits. Antioxidants are crucial because they lessen oxidative stress, a factor in the development of OLP. This can alleviate symptoms and improve clinical outcomes. Studies have shown that antioxidant therapy can significantly reduce pain scores and clinical scores in OLP patients [36]. Systemic vitamin E, a potent antioxidant, has been found to improve clinical parameters in OLP patients, offering a promising adjunctive treatment with minimal side effects [37]. Platelet-rich plasma (PRP), which has antioxidant properties, has also shown efficacy in managing OLP by reducing inflammation and promoting tissue healing [38]. Overall, antioxidants provide a beneficial, non-invasive approach to managing OLP, improving both symptoms and clinical outcomes.

Emerging Therapies

New immunomodulators like Janus kinase (JAK) inhibitors have shown promise in the management of OLP. They work by going after the JAK-STAT signaling pathway, which is a key part of the immune reaction and inflammation that come with OLP [39]. JAK inhibitors, such as tofacitinib, baricitinib, and upadacitinib, suppress both type-1 and type-2 cytokines, reducing inflammation [40]. Case series and reports have demonstrated the safety and effectiveness of these inhibitors in treating various forms of lichen planus, including oral [41]. Ongoing studies are trying to understand the role of JAK inhibitors better in OLP and to optimize their use in clinical practice [42]. Several biologics are currently under clinical trials for the management of OLP. These biologics target specific pathways involved in the inflammatory process of OLP. IL-12/23 inhibitors like ustekinumab and guselkumab have shown promise in treating lichen planus by targeting interleukin pathways [43]. IL-23 inhibitors like tildrakizumab have been reported to be effective in treating lichen planus, particularly in women [43]. IL-17 inhibitors like secukinumab and ixekizumab are being explored for their potential to manage OLP by inhibiting interleukin-17 [18]. These biologics represent a shift toward more targeted therapies, potentially offering better efficacy and safety profiles compared to traditional treatments. Biologics require regular monitoring but are often administered less frequently (e.g., monthly injections). They are also more expensive due to their complex manufacturing process [44]. Gene therapy though is still largely experimental and is an emerging area in the management of OLP. The primary goal is to target the underlying immune response and inflammation that characterize OLP. In an effort to regulate the immune response, gene therapy is designed to modify the expression of particular genes that are involved in inflammation and immune regulation. By targeting cytokines and their receptors, gene therapy can potentially reduce the inflammatory response in OLP. Techniques like CRISPR-Cas9 are being explored to correct genetic mutations or alter gene expression that may contribute to OLP management. Viral vectors, such as adenoviruses, are commonly used to deliver therapeutic genes to the affected tissues [18]. While promising, these approaches are still in the research phase and not yet widely available for clinical use. Current treatments primarily focus on symptom management using topical and systemic therapies.

Supportive Care

Analgesics like paracetamol (acetaminophen) are often used for easing mild to moderate pain and non-steroidal anti-inflammatory drugs (NSAIDs) like ibuprofen for reducing inflammation and pain [18]. Lidocaine is a local anesthetic that can numb the affected area and provide temporary pain relief. Benzydamine hydrochloride is an anti-inflammatory and analgesic mouth rinse that helps reduce pain and inflammation [45].

Future perspectives

The management of OLP is evolving with several promising future perspectives like targeted therapies. Advances in understanding the immunopathogenesis of OLP have led to the exploration of targeted therapies. Biologics, such as TNF-α inhibitors and IL-17 inhibitors, are being investigated for their potential to modulate the immune response more precisely [46]. Research is focusing on improving the efficacy and safety of existing treatments through novel drug delivery systems. For instance, mucoadhesive patches and nanoparticles are being developed to enhance the localized delivery of corticosteroids and other therapeutic agents [7]. PDT is becoming increasingly prevalent as a non-invasive treatment option. It involves the use of photosensitizing agents activated by light to selectively destroy diseased tissues. Early studies show promising results in reducing symptoms and lesion size in OLP patients [47]. Tissue engineering and regenerative medicine approaches are being explored to repair and regenerate damaged oral mucosa. This includes the use of stem cells and bioengineered tissues to restore normal function and appearance [7]. Advances in genetic and epigenetic research are providing insights into the molecular mechanisms underlying OLP. This could lead to the identification of new biomarkers for early diagnosis and personalized treatment strategies [46]. Predictive models for disease progression and treatment response are being created through the application of AI and machine learning. These technologies can help in creating personalized treatment plans and improving patient outcomes. These future perspectives hold promise for more effective and personalized management of OLP, potentially enhancing the quality of life for patients.

## Conclusions

In conclusion, the management of OLP has evolved significantly, with current strategies focusing on symptom relief through topical corticosteroids and systemic treatments for severe cases. Ongoing research is exploring the efficacy of biologics and novel immunomodulatory agents, aiming to provide more targeted and effective therapies. Advances in customized medicine that use molecular and genetic profiling to personalize medicines depending on patient profiles are likely to help future management. Additionally, emerging technologies such as nanomedicine and regenerative therapies hold promise for more innovative and less invasive treatment options. Regular monitoring for malignant transformation remains a critical component across all stages of management, ensuring early detection and intervention. A multidisciplinary approach continues to be essential, integrating the expertise of various healthcare professionals to provide comprehensive and integrated care. In the end, quality of life and patient outcomes are enhanced.
